# Self-medication among the elderly in Iran: a content analysis study

**DOI:** 10.1186/s12877-017-0596-z

**Published:** 2017-09-01

**Authors:** Seyede Salehe Mortazavi, Mohsen Shati, Hamid Reza Khankeh, Fazlollah Ahmadi, Shiva Mehravaran, Seyed Kazem Malakouti

**Affiliations:** 1 Mental Health Research Center, Tehran Institute of Psychiatry–School of Behavioral Sciences and Mental Health, Iran University of Medical Sciences, Tehran, Iran; 2 Department of Aging, University of Social Welfare & Rehabilitation Sciences, Tehran, Iran; 3 University of Social Welfare & Rehabilitation Sciences, Tehran, Iran; 4 Department of Clinical Science and Education, Karolinska Institutet, Stockholm, Sweden; 5 Department of Nursing, Faculty of Medical Sciences, Tarbiat Modarres University, Tehran, Iran; 6 Department of Ophthalmology, Stein Eye Institute, David Geffen School of Medicine, University of California, Los Angeles, Los Angeles, CA USA

**Keywords:** Self-medication, Elderly, Qualitative research

## Abstract

**Background:**

Self-medication is described as the use of drugs without a physician’s prescription to treat self-recognized illness or symptoms, and an important health issue among the elderly. Despite the wide range of different definitions, recognizing all forms of self-medication among older adults, particularly, in developing countries, help healthcare professionals and providers to reduce harmful effects of self-medication. The purpose of this study is to describe the practice of self-medication and its related factors among elderly people in Iran based on the experiences of people who are involved in this phenomenon.

**Methods:**

This qualitative study was conducted using content analysis. Purposive sampling was used to select the participants and continued until saturation. The participants were the elderly, their care-givers, physicians, and pharmacists. Data was collected using semi-structured interviews, and analysis was done using an inductive approach. The theory of planned behavior was used as a framework to explain the role of the emerged factors in the occurrence of self-medication behavior.

**Results:**

Based on the expressed experiences of the participants, factors related to the practice of self- medication among the elderly in Iran fit in these 5 categories***:*** “patient’s attitudes towards disease, treatment, and physicians”, “living with disease”, “unfriendly environments”, “enabling health system”, and “influential others”.

**Conclusions:**

Based on the results of this study, self-medication of the elderly in Iran has commonalities with many countries in regard to over-the-counter medications and complementary and alternative medicine; however, self-medication is also seen with drugs that require a prescription but can easily be obtained from pharmacies. Contributing factors, apart from the elderly themselves, include their families, caregivers, and social circle, the physical environment where they live, and the health system from which they receive services.

## Background

Self-medication is a health-related issue which is currently being discussed and addressed in most countries around the world. Multiple definitions have been presented in different studies, their common point being the use of unprescribed medications to treat symptoms and disease identified by the individual himself or herself. Different forms of self-medication can be found in the literature including use of unprescribed medications, medication refills, medications prescribed to friends and relatives, leftover medications from previous prescriptions, and changing the dosage of prescribed medications. Self-medication can also be in the form of using pharmaceutical medications, traditional and home remedies, and supplements [1–4].

Studies have pointed out multiple problems related to self-medication which includes increased risk of adverse drug reactions, drug interactions, drug resistance, and even sudden death in certain cases. These eventualities are partly attributable to prescription drugs used on an individual’s own initiative and partly to misuse or abuse of OTC medicines [4–7]. Nonetheless, self-medication is not always considered a negative type of behavior. When self-medication is practiced using over-the-counter (OTC) medicines, it is considered an important element of self-care emphasizing the role of each individual in their own health [1–3, 5]. Therefore, it seems that when benefits of self-medication are discussed, it refers to medicines that an individual is allowed to use without consulting a health professional [5]. As described by the World Health Organization, these products have certain specifications, they are produced for this very purpose, they even have different packaging, and they are provided with patient information leaflet [1] which can lead to harm if overlooked.

Of note, the list of OTC medicines and their provision methods vary by country, and their access and consumption patterns depend on the nation’s health system and regulations. In some countries, such as the United States of America, OTC medicines are sold directly to the consumer in drugstores and general stores. In the United Kingdom and many other European countries, they are only available in drugstores where they are dispensed at the discretion of the pharmacist, and only small amounts of low doses may be available in general stores [5]. These differences may impact the prevalence and forms of self-medication that occur.

Self-medication may be practiced at any age, but when it comes to the issue of treatment and medications, special attention is given to the elderly as a special group, because various studies have found them to be the largest consumers of medicines in most countries, and Iran is no exception to this fact [8, 9]. One reason for the higher rate of medication use and self-medication among the elderly is the higher risk of many diseases at older age. A large proportion of the elderly population suffers from ailments such as cardiovascular disease, diabetes, and cancer. Also, comorbidity of chronic diseases is common in this age group, and can lead to increased use of medications [9, 10]. On the other hand, age-related changes in pharmacokinetics and pharmacodynamics make drug-related issues in the elderly even more complex than other age groups [9], and they need to be addressed with major attention.

Multiple studies have addressed self-medication around the world, and multiple causes have been identified. Causes include the high costs of treatment and insufficient insurance coverage, and therefore, many people who cannot afford physician’s fees will resort to self-medication. Other causes mentioned in the literature include avoiding loss of work-time, distrust in health personnel, long waiting times for seeing a physician, having previous experiences with the disease, and underrating disease [4, 6, 11, 12].

Available studies on self-medication in Iran are descriptive and focus on the prevalence and reasons for self-medication, diseases associated with self-medication, and common medicines used in this regard [7, 13–15], few have addressed the issue among the elderly [16]. Also, qualitative studies identifying associated factors based on the experience of consumers and involved individuals have rarely been done. Since the provision and consumption of medicines in Iran is quite different, qualitative studies on this issue are essential specially for effective interventional planning.

Unlike many other countries, in Iran, only pharmacies are allowed to sell prescription and non-prescription medicines [17], although there are illegal forms of access as well. Pharmacists and physicians have no access to patients’ medical records, and in many cases, no record exists. Despite pharmacists’ knowledge of the OTC drug list, non-OTC drugs are provided to customers without a prescription at many pharmacies [17]. Medications are low-priced in Iran [18] and easily accessible. In addition, although 90% of the population is insured, the out of pocket share is significant and can impose heavy financial burdens on patients [19]. As a result, many patients opt to purchase non-prescribed medications rather than seeing a physician. All these facts create a different situation in Iran.

Overall, a variety of factors can be involved in the practice of self-medication and predisposing factors may vary by population and culture. Also, in-depth studies in this area are very limited in Iran. Therefore, in light of the unique circumstances of its health care system, this study aims to achieve a comprehensive understanding of self-medication practices by elderly people in Iran and its determinants based on the experiences of stakeholders including the elderly, their caregivers, physicians, and pharmacists.

## Methods

### Study design

This is an inductive qualitative content analysis study of self-medication among elderly people in Iran. The researchers performed an in-depth direct analysis of stakeholders’ experiences. Any prejudgment and expression of personal opinions were avoided during data collection and analysis. In this regard, the applied strategies included memoing, member check, interviewer training and engaging experts in qualitative studies in the research team. The results are presented as codes, subcategories, and categories using an inductive approach [20], which were then further organized based on the constructs of the theory of planned behavior (TPB) to allow for the interpretation of what influences self-medication practices by elderly people in Iran.

### Participants and study setting

Participants were selected through purposive sampling from stakeholders who were believed to be the most information rich regarding their experiences with self-medication. Selection of study groups was based on the objective of the study, and in light of the research question, study samples included groups of elderly people, their caregivers, physicians, and pharmacists.

The inclusion criterion for elderly participants was having a history of self-medication. This was determined through review of their medical history by a physician and finding at least one non-OTC item which was consumed without a prescription. Enrolled caregivers were in charge the elderly who practiced self-medication, lived with them, and were fully aware of their life situations. The inclusion criterion for physicians and pharmacists was to have encountered elderly patients.

All study participants were residents of Tehran, consented to participation, had favorable communication skills, and were interested in sharing their experiences. As agreed with the participants, interviews with the elderly and their caregiver’s were conducted at their home or the hospital where they were hospitalized, and interviews with physicians and pharmacists were done at their workplace.

The endpoint for sample selection was reaching data saturation regarding self-medication [21]. At this point, additional information obtained from the 3 final interviews did not vary the categorizations.

### Data collection and analysis

The data for this study was collected between July 2015 and May 2016 through in-depth semi-structured interviews with the participants. Interviews were conducted by the first author (SSM) who has experience in research on the elderly and trained for conducting qualitative interviews. In addition to interviews, we used available documents and evidence such as the list of OTC medicines and related regulations as well as prescription records, their medicine bags, and medicines they had stored at home. Elderly participants were approached at the residence where a researcher and a general practitioner examined the contents of their medicine bags and their prescription records, obtained a medication history, and determined their eligibility for study inclusion.

Interviews with the elderly started with their experience about self-medication, and according to the interview guidelines, general open-ended questions were asked saying “Think of an instance when you were feeling unwell or in pain. What did you do to alleviate this feeling?” or “Describe an instance when you were using medicine without consulting a physician.” Then, depending on the context of the responses, the interviewer continued with exploratory questions such as “Can you please give an example?” or “Could you explain more?” to clarify concepts for the researcher and participants. The duration of the interview ranged between 45 and 90 minutes depending on the willingness and situation of the respondent. Since certain issues could be beyond the scope of the interview, respondents were given the opportunity for an informal conversation at the end of the interview by asking them “Would you like to add anything else?” Each respondent had 1–3 interviews, as needed. Additional interviews were scheduled when participants still had more to say or the preliminary analysis (transcription and coding) indicated a need for obtaining further information. In the latter case, interviews were kept short and limited to specific questions. Other participants were enrolled based on data direction.

**Fig. 1:**
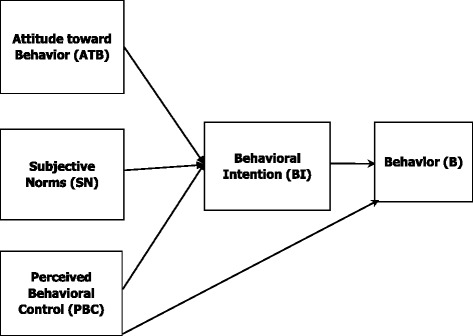
Theory of planned behavior framework

After each interview, audio files were listened to several times and verbatim transcriptions were prepared. Each entire interview was considered as an analysis unit. The transcribed script was read several times to become familiar with the context, after which, the meaning units were identified. The meaning units were then condensed according to their content and context. Then, the condensed meaning units were abstracted and labeled with a code (Table 1). Which were then compared based on differences and similarities and sorted into sub-categories and categories [20] (Table 2). Finally, the results were organized into the framework of TPB. According to TPB, behavior is linked to an individual’s intention and ability, which are discussed as behavioral intention (BI) and perceived behavioral control (PBC) in this model. Figure 1 illustrates the association between TPB constructs and their role in the initiation of a given behavior [22].

**Table 1:** Examples of meaning units, condensed meaning units, and codes from content analysis of the participants’ experiences

Meaning unit	Condensed meaning unit	Code
The problem is that we didn’t know who to see for mom’s problem. And there was no one to advise us. We kept going to different doctors others’ recommended.	Going to different doctors recommended by others	Choosing physicians recommended by one’s social circle
The doctor prescribed me a bag of drugs without saying a word. So, what am I supposed to do with these? I get confused	Prescribing without talking to the patient	Failure to provide instructions for using the drug
I go to the pharmacy myself and name what I want or I take the packaging. Even if I don’t know the name, I ask the pharmacists. They give me any medicine I want.	Pharmacists give me any medicine I want	Purchasing prescription medication from the pharmacy without a prescription

**Table 2:** Examples of codes, sub-categories and categories from content analysis of participants’ experiences

Theme	Self-medication				
Categories	Enabling health system			Patient’s attitudes towards disease, treatment and physicians	
Subcategories	Confusion in choosing a care provider	Patient confusion after the visit	Unregulated availability of medication	Underrating disease	Considering medication safe and effective
Codes	Choosing physicians based on their popularity	Patient unawareness about disease and treatment processes	Purchasing prescription medication from the pharmacy without a prescription	Believing that certain mild diseases do not require a physician	Not counting herbal and traditional medicines as medication
	Choosing physicians recommended by one’s social circle	Failure to provide instructions for using the drug	Pharmacies prescribing medication for patients	Believing that the disease will resolve spontaneously	Assuming vitamins to be good for everyone
	Choosing physicians recommended by other patients	Failure to provide instructions for dealing with side effects	Obtaining medication in amounts more than necessary	Relating disease to age	Assuming painkillers to be safe

### Trustworthiness of data

In the process of this study, we adhered to the recommended criteria for the assessment of qualitative data [20, 23, 24]. The principal investigator dedicated about one year for the collection and analyses of the data and was deeply involved with data during this time to ensure credibility and acceptability of data. In addition, triangulation was used for data collection. We also used member checking, expert check, and peer check to ensure the credibility of the results. For this purpose, all interviews were coded separately by two of the researchers (SSM and MS). Cases of difference were discussed at team meetings. Validation during interviews was accomplished by restating or summarizing the information and asking the participant to determine accuracy. Also, certain coded interviews were reviewed by participants to ensure the researchers’ interpretation of the data. To perform external audit, the data was reviewed by an independent expert, who has extensive experience in qualitative research. To establish confirmability of the results, research processes are described in detail to provide others with the opportunity to follow up the research. To improve transferability, the demographics of the participants and the topic of interest are described in detail to allow the reader to decide about how to use the results.

### Ethical considerations

This study was approved by the Ethics Committee of the Iran University of Medical Sciences. The study purpose and procedures were fully explained to target individuals before each interview, and only those providing informed consent were enrolled in the study. Participants were assured of confidentiality and anonymity of the information provided, and they were free to withdraw from the study at any point. Participating elderly who had presented for treatment were assured that their non-participation or withdrawal would not affect their treatment or care.

## Results

The participants of the study were 21 people, 12 women and 9 men, who took part in a total of 25 interviews. Participants included 10 elderly people 60 years of age and older, 3 caregivers, 3 pharmacists, and 5 physicians. The mean age of the participants was 57.78 ± 14.1 years, and the mean age of the elderly was 69.6 ± 8.4 years. Among participants, 16 were married, 3 were widowed, 1 was single, and 1 was divorced. Two of the participating elderly were illiterate, 3 had elementary education, 3 had completed middle school, 1 had a high school diploma, and 1 had a bachelor’s degree.

In this study, respondents mentioned the following forms of self-medication:

1. OTC medications
2. Complementary or alternative medicines
3. Non-prescribed prescription medications recommended by the selling pharmacist
4. Prescription medications purchased using expired and non-refillable prescriptions
5. Leftover drugs from old prescriptions
6. Prescription medications offered by family and friends.

For example, elderly participant No. 3 stated: “I get it from the pharmacy myself. Some doctor prescribed to me some time ago, and now I just take the packaging and tell them that’s what I want. Sometimes I ask them at the pharmacy. I tell them my problem and they tell me which medicine is good and they give it to me.”

Caregiver No. 2: “Her friend told her that she was taking chlordiazepoxide, it’s good, and she was so calm. Now she’s started taking it too.”

Elderly participant No. 2: “I had some myself. A doctor had prescribed it to me once. I still had some at home. So I started taking it. ”

Self-medication is sometimes the first line of treatment, and the patients seeks medical advice only after self-medication fails, while in other cases, self-medication is practiced as the substitute treatment.

Our study participant’s experiences about self-medication and its determinants were grouped in 5 categories: “patient’s attitudes towards disease, treatment, and physicians”, “living with disease”, “unfriendly environments”, “enabling health system” and “influential others”; each had their own subcategories. These results are summarized in Table 3 and discussed in the following sections.

**Table 3:** Categories and subcategories extracted from the qualitative content analysis of participants’ experiences

Subcategories	Categories	Theme
Underrating disease	Patient’s attitudes towards disease, treatment and physicians	Self-medication
Mistrust in physicians		
Considering medication safe and effective		
Fear of going to a doctor		
Severity of symptoms	Living with disease	
Prolonged and persistent symptoms		
Urgent need for treatment		
Overcrowded clinics	Unfriendly environments	
Unsuitable clinics for the elderly		
Unsuitable environment for elderly transportation		
Drug accessibility	Enabling health system	
Unregulated availability of medication		
High doctor’s fees		
Cheap medicines		
Confusion in choosing a care provider		
Weak patient information system		
Patient confusion after the visit		
Interfering friends	Influential others	
Family defining the treatment path		
Active caregiver involved in the treatment		
Others patients’ success stories		

### Patient’s attitudes towards disease, treatment, and physicians

In some cases, elderly participants turn to self-medicate because they believed that their diseases were self-limiting, did not take their disease seriously, or they found an intervenable and simple justification for their problem with the logic that the disease was unvarying. Some of them indicated that age-related complaints are common among their peers and most of their symptoms are caused by the aging process, therefore visiting a doctor is useless.

For instance elderly person No. 2 said: “Ten years ago my doctor said I had high blood sugar, prescribed a pill and said take one in the morning and one at night. I didn’t take it seriously and took one instead of two pills. I told myself I’d work and burn the extra sugar by working. Then my disease got severe.”

Elderly participant No. 8: “Some diseases, like the heart, are no joke. You have to see a doctor right away. You have to take them seriously. You can’t just do something about it yourself. But things like joint pains, headaches, and colds don’t need a doctor. I can somehow handle it myself.”

Caregiver No. 2: “She resists going to the doctor and thinks that’s how it is, won’t get better, it’s because of her age and has to bear it. She says what’s going to come of going to the doctor?”

On the other hand, elderly’s perception of drugs and their belief that certain medicines such as pain killers, vitamins, home and herbal remedies, and even certain antibiotics are harmless and effective leads them to use them groundlessly. In this regard, pharmacist No. 1 said: “A lot of times, they think they’d get better if they keep taking the same drug. We keep telling them that it has a certain course of treatment, and they may have side effects if used more than prescribed. If I refuse to sell it, they’ll go get it from the next pharmacy.”

Physician No. 3 said: “Usually, patients have already used home and herbal remedies and any medications that they perceive as effective when they come see us, especially with colds and digestive problems. They take herbal teas and extracts. They don’t count them as medicine, they think they’re safe, and they use them.”

Elderly No. 2: “I believe in herbals because they’ve worked for me. If they didn’t work, I wouldn’t. For example, I didn’t know yellow goatsbeard is good for the heart. My brother told me, I tried it, and it was good. It’s best that we old people medicate with these herbs. They’re harmless and all.”

Our data showed that negative attitudes toward visiting a physician leads to self-medication. For elderly participants, these negative attitudes ranged from psychosocial issues such as mistrust, fear of hospitalization and surgery, fear and anxiety of being diagnosed with medical conditions (particularly terminal illnesses) or adding more to their medications, fear of medical interventions due to previous problems and complications and also logistical issues. The logistical concerns for visiting a doctor include: lengthy waiting times at the doctors’, long interval between identifying a need for specific tests and treatments and actually receiving those services, and lack of support from their caregivers or families.

Elderly participant No. 1 expressed: “I don’t go to the doctor’s if I can help it, and pain doesn’t bother me, because doctors are like construction workers. It’s the same with doctors. They keep sending you for a checkup here and a checkup there. They’re bound to find something eventually. Now the doctor tells me to get an angio, but my mom got a bruise in her leg when she did hers. It was hard for my sister too. She couldn’t walk at all. I’m too scared to do it.”

### Living with disease

According to our data, self-medication among older adults is influenced by high pain intensity, severity of other symptoms, and urgent need for treatment of sudden symptoms. In addition, prolonged and persistent symptoms after receiving (ineffective) care from providers also leads to all types of self-medication. For instance, elderly participant No. 8 recounted: “I had a kidney stone and the pain was getting too much. Excruciating! I even went to the doctor’s but the drugs didn’t work. I did anything to ease the pain. I took any painkiller.”

Caregiver No. 1: “When she has unbearable symptoms or severe nausea and vomiting that are too disturbing for her, she will do anything anyone recommends because she can’t bear it. Some neighbor might suggest it’s something she ate, and that she should take some herbal or other, and she listens.”

### Unfriendly environments

The experiences of the participating elderly indicated that environmental factors can prevent seeing a doctor and set the grounds for self-medication among the elderly.

In this regard, caregiver No. 2 stated: “If you want to take her to the doctor, you have to get a cab, and it costs too much. If you want to walk, just look at the sidewalks. Do you think they’re suitable for the elderly? It’s all troughs and bumps. If you want to take public transportation, she can’t get on. That’s why it’s totally difficult and you can’t go to the doctor’s too frequently. Not going to the doctor has effects too. For example, her friends told her they were taking chlordiazepoxide, it’s good, and they’re calm. And now she has started taking it too.”

Caregiver No. 1: “We can’t move mom. It’s really hard. She had a hand skin problem. I had to get a photo and take it to the doctor. I couldn’t take mom because there was a staircase to the office and no elevator. Besides, the office is crowded and my mom can’t wait a turn on account of her condition. She can’t sit long, and if the waiting time is too long she needs to use the restroom and most offices don’t have a suitable one for the elderly.”

### Enabling health system

Certain factors relate to the health care provision system in Iran. Medicines are available to patients with no limitation. All participants had the experience of simply going to the pharmacy and purchasing any medicine they needed with or without a prescription. This method is easier and more cost-effective for them than going to a physician’s office and spending extra time and money. Moreover, the low price of medicines in Iran compared to many other countries makes it easier to access drugs. Also, patients have to pay a high share of the costs of office visits and treatment, and all these issues set the grounds for self-medication.

In this regard, elderly person No. 3 stated: “I go to the pharmacy myself and name what I want or I take the packaging. Even if I don’t know the name, I ask the pharmacists. They give me any medicine I want. I get to save the physician fee, and I can get my apprentice to go get it for me if I’m busy.”

Pharmacist No. 2 indicated that: “A packet of cold medicine or even antibiotics is cheaper than bread. This means medicine is cheaper than basic needs. Patients see if they go see a doctor, they have to pay the fee, it’ll cost them more. So they’ll go get it from the pharmacy. And there is a very bitter joke between pharmacists that we have close to 3000 OTC medicines in Iran, and the list practically includes all drugs.”

Hanging in the balance in the health system is another contributing factor to self-medication. When individuals feel unwell, they do not know who to consult, and they are faced with a long list of specialists; eventually the choice is up to them. Almost all participants recalled having seen several physicians for the treatment of the same disease. This is while most specialists they chose were irrelevant to their problem, and seeing them was a waste of time and expenses. Eventually, many cases resorted to self-medication to alleviate their disturbing symptoms.

In this regard, caregiver No. 3 stated: “The problem is that we didn’t know who to see for mom’s problem. And there was no one to advise us. We kept going to different doctors others’ recommended; it was no use. During this time, she tried everything anyone suggested.”

The lack of any record regarding patients’ medical history and medications is another issue in the health care system. Physicians and pharmacists have no knowledge of the drugs used by patients and provide them with any kind of medicine. In this regard, pharmacist No. 1 stated: “As a pharmacist, I don’t have their medication history. Since I don’t have any access to the other drugs they’re using, there’s not much I can do. So I give patients the drugs they want. If I don’t, they’ll get it at the next store.”

Receiving insufficient information at the time of visiting a doctor is another predisposing factor. In many cases, the physician does not provide enough information about the course of the disease, instruction about prescribed medications and their possible side effects, and thus, the patient feels confused even after seeing a physician. Since they are undecided about whether they should return or get a refill, they resort to self-medication.

In this regard, elderly participant No. 5 stated: “The doctor prescribed me a bag of drugs without saying a word. So, what am I supposed to do with these? What am I supposed to do when I finish these? I get confused.”

### Influential others

People around the elderly patient play an important role in self-medication. Elderly people’s families and caregivers can have a strong influence on how they deal with therapy and use of medications because the elderly often rely on them for seeing a physician and managing their medications. On the other hand, friends and neighbors exert their influence by sharing their experiences. In many cases, people who have experienced the same illness recount their experiences and influence the elderly directly or indirectly with their suggestions. For instance, caregiver No. 2 said: “The lady I care for started using a drug after a friend of hers recommended it, because she said it worked for her. That’s how she got started. Or her son brings her medications from abroad. She goes to the park and someone tells her what their doctor gave them for their pains, and she goes to the pharmacy and gets the same thing.”

### Applying the TPB

**Fig. 2:**
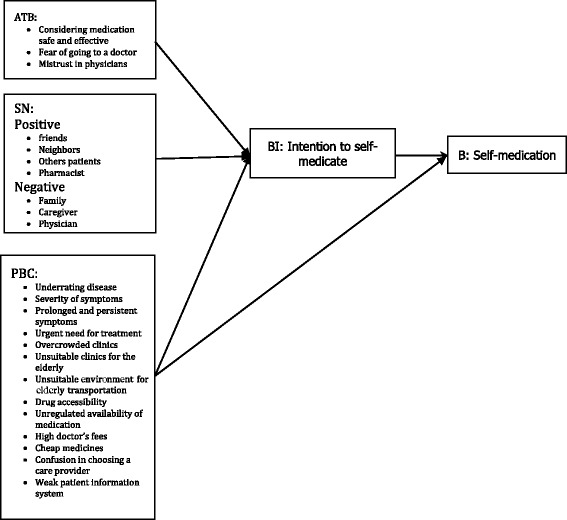
Description of the self-medication behavior in elderly people in Iran using the theory of planned behavior framework

As presented in Fig. 2, all codes, sub-categories, and categories extracted from data analysis were organized in accordance with the constructs of the TPB (Fig. 1) and classified as attitude toward behavior (ATB), subjective norms (SN), PBC and BI.

## Discussion

Based on the results of this study, self-medication among elderly Iranians occurs under circumstances where the individual is not the only person who has a role in it. While certain factors are related to the elderly themselves, the environment they live in, the health care platform, and even the families, caregivers, and other people around them play an important role in their treatment process and their decision to self-treat. A common problem in Iran is using non-prescribed prescription medications. Our data showed that the unauthorized use of prescription medications in Iran is not a rare or isolated self-medication approach. Almost all participants pointed to the easy access of Iranian adults to such medications that are obtained from local pharmacies. Use of old/expired prescription medications, unauthorized refills and receiving them from friends, and family are also common.

Although the present study had an inductive approach and the presented concepts emerge from the data, results can be explained within the frameworks of certain theories and models in health behavior studies. Compared to available theories, the extracted categories in this study seem to be consistent with the TPB, which is well-known among health behavior theories and has three constructs: attitude toward behavior (ATB), subjective norms (SN) and perceived behavioral control (PBC) [22, 25, 26]. Collectively, these three constructs can explain 50% of the variance in an individual’s intention to perform a certain behavior [25]. Therefore, we found this theory most suitable for the assessment of self-medication which is a multidimensional behavior with unique complexities.

Our study results indicated that patients’ attitudes, beliefs, and perceptions about disease and different ways of dealing with it are a major determinant of patients’ behaviors. When patients believe there is no need to see a doctor, they definitely turn to self-medication. This is while sometimes a symptom such as fever is considered ordinary and simple by patients, but can be a serious sign of disease for the elderly [27]. In addition, in many cases, they attribute their symptoms to age, and they assume nothing can be done about it, so they need to bear the situation or somehow manage it themselves. Given the low levels of health literacy among the Iranian elderly population, this was an expected finding [28] and it points to the need for public awareness and simple patient-friendly education programs through media and health care centers.

The emerged concepts from this section are consistent with the PBC construct in TPB which refers to people’s perceptions of their ability to perform a given behavior [26]. This construct underscores the presence or absence of facilitators and barriers to performing a certain behavior and their level of impact on the ease and difficulty of performing the behavior. In the presence of control beliefs about the facilitators, the individual’s perceived ability to perform a given behavior is enhanced [22].

Facilitators of self-medication in the elderly include situations when they have mild or tolerable symptoms or conditions that can be attributable to older age. In these cases, individuals believe themselves to be self-efficient and capable of handling the situation on their own. Therefore, by taking into factor the effect of patients’ attitudes about disease on the intention to self-treat, and the subsequent behavior, this construct can be used to design behavior change interventions to prevent self-medication when there is need to refer to a physician.

Based on the results Patients believe many medications such as sedatives, supplements and herbal medicines are safe, and thus, they consume these products carelessly. Self-medication with medicinal herbs and herbal medicines is common practice. The belief is that these medications are useful or at least there is no harm in using them. A positive attitude of the elderly toward the effects of self-medication with these groups of medications on their health and diseases is one of the important factors leading to self-medication behavior which is known as ATB in TBP. ATB is defined as positive or negative reactions or evaluative affects about a behavior which display as feelings, beliefs, and even behaviors. In other words, when people believe a given behavior will lead to valuable outcomes, they will develop a positive attitude toward that behavior, and vice versa. As a result of this positive attitude, such medications are consumed without any consideration to precautions, especially that they are often combined with pharmaceutical products. Designing interventions that aim at correcting this false belief can reduce the risks associated with the improper use of sedatives, supplements and herbal medicines.

Non-prescription medications used for self-medication often come with patient information sheets, but the main challenge in Iran is that, unlike many other countries, reading them is not common practice [29]. Therefore, in addition to evaluation and planning for public awareness and education about medications, there is needed to implement culture changes in using drug labels and information sheets.

Based on the participants experiences distrust in physicians is another factor that arises from having a history of failed treatment, misdiagnosis and the belief that diagnostic procedures are unnecessary and prescribed by physicians mainly for their own financial gain. Distrust in medical services has been mentioned by Sawalha and Shaghaghi et al. [11, 30]. In these situations, the elderly find it useless and unnecessary to consult physicians, and as suggested by the ATB construct in TPB, this negative evaluation or the resulting negative attitude can set the stage for the intent to self-medicate in order to manage disease symptoms.

Most of the elderly resisted going to a physician as far as they could. The refusal is sometimes due to fear of being diagnosed with a dangerous illness or the possibility of a serious new problem. Although studies suggest that fear of death decreases in old age compared to other age groups [31], they wish to die at home, and part of their fear for referring to a physician may be for this very reason. The elderly are more likely to think about death [32]; therefore, it is important for them to have coping skills to deal with fear of death. This issue has received little attention in Iran, but the integration of elderly care into the health system network has provided the right opportunity for planning and transferring such skills. In terms of life-threatening illnesses, developed countries have been providing palliative care to patients and their families for many years [33]. These services are yet not available in Iran [34]. In light of the growing elderly population, appropriate planning for such services seems necessary.

Other factors expressed in this study concerned situations when the patient struggled. Practicing self-medication in acute situations that are unbearable for the elderly is another good example of the function of the PBC construct in the TPB model. Even those who closely follow their physicians’ orders might do anything they can when they have acute and agonizing symptoms, and they may turn to self-medication while they wait for the chance to see their physician, or even afterwards when their symptoms do not resolve. Under these circumstances, the elderly resort to a solution to alleviate their symptoms until the physician arrives or the situation becomes bearable. From another point of view, the circumstances facilitate performing self-medication. Presence of severe symptoms and failure to resolve them were the most common reasons for self-medication. In the study by Pineles and Parente as well [35].

In Iran, an additional problem is lack of urgent care services. There are cases with non-life-threatening conditions who do not feel the need to present to the emergency room, but arranging an early office visit is not possible either, and urgent care seems the right services for them [36]. There is need for establishing urgent care services in Iran through proper evaluation and planning.

Another expressed issue in our study, which fits as a PBC construct, concerns complicated situations due to chronic disease comorbidity. Self-adjusting the dosage, discontinuing their use, forgetting certain medications, and self-medication, are among other issues that arise from multimorbidity and multiple drug consumption. This result is consistent with previous studies [37]. In most cases, they had to consult several physicians for multiple chronic conditions, therefore, they tried to self-manage any newly developed symptom or problem so that they would not need to consult a new physician and receive additional medications. In such circumstances, the elderly’s evaluation points to a more complicated situation and greater risk of side effects due to multiple drug consumption. This negative evaluation results in the formation of the ATB construct and a negative attitude toward returning to the physician, and practicing self-medication to avoid exposure to greater risk. Similarly, factors related to the ATB construct in the study by Widayati et al. included a range of concerns about side/adverse effects [26].

In many cases, the physical environment is unsuitable for the elderly and prevents them from seeing a doctor on their own when they feel the need. This factor deprived them from available health services and they resorted to self-medication at home. While special assistance is available for the handicapped and the elderly in developed countries, public transportation in Iran has not considered the special needs of incapacitated individuals. In addition, sidewalks and pedestrian passageways are not proper or safe, and clinics lack disabled facilities. Concepts within the category of “unfriendly environments”, as described here, facilitate performing self-medication among elderly through a mechanism consistent with the PBC construct.

New theories of disability also suggest that unsuitable environments can hinder the utilization of health care services, and thus, the individual resorts to self-managing the disease and its symptoms; this problem could be avoided if the given individual was in a well prepared and supportive environment [38]. Should we take the viewpoint of previous disability theories, there would be the fact that individuals are debilitated at older age and depend on others to see a physician, and the issue could be categorized as a patient-related factor. In agreement with Hayati et al. [39], we found a relationship between over-crowded and unsuitable offices and self-medication. This factor is also consistent with the PBC construct and concept and is predictive of the intent and performance of self-medication. Wen et al. also suggest that environmental factors can contribute to self-medication. Although the environmental factors they discuss may differ from those found in this study, they agree that factors such as easy access to medicine along with suggestions by friends and peers are important factors [6].

The setting of the health care services can play a facilitating role in self-medication as well. Factors such as easy access to low-priced drugs, high physician fees, and other issues in this category make the health system and its components consistent with the PBC construct in the TPB model. When treatment does not follow a specific process and patient information is not recorded in a unified system, the patients are free to go to any physician and pharmacy and ask for the medications they need. Patients referring to pharmacies are unaware whether the medicine they are asking for is an OTC drug or requires a prescription. The lack of awareness in our country is probably because, unlike other countries, OTC drugs in Iran are not sold off the shelf and they are treated similar to other drugs. In any case, the provision manner of OTC medications along with unawareness about the list of such medications complicates the problem. In this regard, the role of pharmacies which sell off-list items despite their knowledge of the list of OTC drugs should not be overlooked. In this process, most medications are practically available as simply as OTC drugs are. This issue has been discussed in the study by Sahebi et al. as well [14].

An additional issue expressed by our participants is the low price of drugs in Iran. Studies suggest that this is because 95% of them are manufactured domestically [40] and unlike some other countries, prices are regulated by the government and not the market. The government has policies for price control to maintain prices low and fixed nationally [41]. Therefore, drugs are easily obtained and large amounts are accumulated at home; this leads to unrestricted access of the general public to drugs. The results of some other studies support the important role of drug availability in self-medication [13, 18, 42]. Similar to the study by Armitage et al., [43] the concept of cost of medications was considered a factor of PCB. Access to medications has been classified among control beliefs in the study by Widayati et al. as well [26].

Another concern within the health setting in Iran is that, although most people have health insurance, out-of-pocket percentages are high, and health expenses can impose a heavy financial burden. As a result, people may settle for obtaining medication without a prescription rather than seeing a doctor. Mehrdad has discussed this matter in his description of the health system in Iran [19]. In the study by Sahebi et al. in Tabriz, more than a quarter of the individuals who practiced self-medication stated cutting costs to be the reason [44]. Afshari et al., Jafari et al., and Jain et al., pointed to high physician fees along with other factors such as having a history of disease [13, 45, 46]. This issue, like other facilitating features of the health system, can predispose an individual to perform self-medication directly or indirectly through mechanisms described for the PBC construct.

When a patient consults a physician but does not receive sufficient information about the treatment process, they are less likely to follow the physician’s instructions, and in many cases, they make decisions themselves. In this regard, physicians’ work load and busy clinics, especially in public settings, should not be overlooked. Physicians participating in this study stated that their busy clinics did not leave enough time for them to discuss matters with each patient.  In addition to the mentioned issues, the people who influence the elderly can have a substantial effect in the treatment process. It appears that this influence is even stronger when herbal and traditional medicines are concerned, which is due to the misconception about their safety. Family and caregiver support for receiving medical care is mentioned in other countries as well, especially for elderly who are incapable of living independently [45]. This finding is consistent with the SN construct in the TPB model. SN results from normative beliefs which are influenced by the judgment of one’s social circle and significant others. In this study, the positive attitude of peers and friends toward self-medication and suggesting the use of non-prescribed medications acts as a facilitator for the intent to self-medicate. On the other hand, family members’ concerns with the risk of adverse effects on account of self-medication and exacerbation of the disease by delaying seeing a doctor can have a constraining effect. Armitage et al. suggest that health experts and families exert subjective norms most strongly [43]. Friends and family were also found responsible for positive normative beliefs for using non-prescribed antibiotics in the study by Widayati et al. as well [26].

In Iran, however, the elderly have low levels of literacy [47], and consequently, they use books and internet less often; therefore, their family, caregivers, friends, and peers become their source of health information [13, 15, 39]. In this scenario, the social circle plays a key role in defining their health behavior.

In conclusion, as presented in Fig. 2, the concepts derived from this study seem to be consistent with the constructs with the TPB model, and can be used in designing further analytical and interventional studies. The results of this study can also be interpreted through other health behavior models. For example, among models concerned with geriatric health, the constellation of factors determined through this study seems to be in line with the ecological model [22].

According to this model, determinants of health behavior can be fit into interacting categories that form a given behavior. Each one of these factors may belong to the intrapersonal, interpersonal, organizational, community, and public policy categories. Therefore, if self-medication is considered an elderly patient’s health-related behavior, the behavior is formed by patient’s attitudes and living in pain in the intrapersonal level, influential others in the interpersonal level, service providers in the organizational level, improper physical environment in the community level, and enabling health setting in the public policy level.

### Limitations

This study had certain limitations. Since data was collected using participants’ previous experiences, similar to other studies of this kind, there is the probability of recall bias. Since recall bias is more likely among older participants, focus was placed on their more recent experiences to reduce this bias, and where necessary, data was completed and verified through family members. Another limitation is that people may not be willing to admit having behaviors that may appear to be improper by others. This tendency can increase the social desirability bias, and the most important measure to reduce this bias was to gain their trust. Other approached used to reduce this type of bias included asking indirect questions so that the behavior would not seem unjustifiable or unacceptable.

## Conclusion

The elderly in Iran practice different forms of self-medication that involve prescription, non-prescription and herbal/complementary medicines. Determinants of performing self-medication behavior, as identified in this study, include a variety of factors that are consistent with the constructs forming behavioral intention and behavior in the TPB model. These include patient’s attitudes, living with disease, unfriendly environments, enabling health system, and influential others.

## Abbreviations

ATB: Attitude toward behavior
BI: Behavioral intention
OTC: Over-the-counter
PBC: Perceived behavioral control
SN: Subjective norms
TPB: Theory of planned behavior
